# RETRACTED ARTICLE: Sudden cardiac arrest under spinal anesthesia in a mission hospital: a case report and review of the literature

**DOI:** 10.1186/s13256-018-1648-5

**Published:** 2018-05-24

**Authors:** Bamidele Johnson Alegbeleye

**Affiliations:** Department of Surgery, Banso Baptist Hospital, P.O Box 9, Nso-Kumbo, Northwestern Region, Cameroon

The Editor-in-Chief has retracted this case report because of concerns about patient consent to publish. The content of this article is no longer available online to protect the privacy of the patient. The author has not responded to correspondence from the publisher about this retraction.

